# A simple new method to determine leaf specific heat capacity

**DOI:** 10.1186/s13007-025-01326-3

**Published:** 2025-01-24

**Authors:** Jiayu Zhang, Elias Kaiser, Hanyi Zhang, Leo F. M. Marcelis, Silvere Vialet-Chabrand

**Affiliations:** Horticulture and Product Physiology, Department of Plant Sciences, Wageningen University & Research, Wageningen, Netherlands

**Keywords:** Heat capacity, Energy balance, Thermal imaging, Dynamic environment, Natural variation

## Abstract

**Background:**

Quantifying plant transpiration via thermal imaging is desirable for applications in agriculture, plant breeding, and plant science. However, thermal imaging under natural non-steady state conditions is currently limited by the difficulty of quantifying thermal properties of leaves, especially specific heat capacity (C_p_). Existing literature offers only rough estimates of C_p_ and lacks simple and accurate methods to determine it.

**Results:**

We developed a non-invasive method to quantify k (the product of leaf thickness (lt), leaf density(ρ), and C_p_), by fitting a leaf energy balance model to a leaf temperature (T_leaf_) transient during and after a ~ 10 s light pulse. C_p_ was then estimated by dividing k by lt*ρ. Using this method, we quantified C_p_ for 13 horticultural and tropical plant species, and explored the relationship between C_p_ and leaf water content, specific leaf area and T_leaf_ response rate during the light pulse. Values of C_p_ ranged between 3200–4000 J kg^−1^ K^−1^, and were positively correlated with leaf water content. In species with very thick leaves, such as *Phalaenopsis amabilis*, we found leaf thickness to be a major factor in the temperature response to a short light pulse.

**Conclusions:**

Our method allows for easy determination of leaf C_p_ of different species, and may help pave the way to apply more accurate thermal imaging under natural non-steady state conditions.

**Supplementary Information:**

The online version contains supplementary material available at 10.1186/s13007-025-01326-3.

## Background

Infrared thermography is a non-destructive and sensitive method for capturing the surface temperature distribution of an object based on its infrared radiant energy [9, 37]. With the development of imaging technologies, thermography has attracted attention in agriculture, where it is mainly used to monitor (a-) biotic stress incidence, and to provide irrigation decision support [14, 18, 33]. In plant physiology and plant breeding, it is used in high-throughput phenotyping platforms to screen for differences in water use efficiency and stomatal regulation [28, 29, 32]. Additional thermographic work relying on the leaf energy balance was used to infer physiological parameters such as stomatal conductance (g_sw_) and transpiration rate (E) [23, 38, 39]. Most studies rely on steady-state equations of energy balance under stable environmental conditions, based on energy absorption and loss between plant-environment interactions, ignoring the importance of the thermal properties of the leaf itself. However, thermal imaging applications are also increasingly used under non-steady-state conditions [38, 39], where understanding leaf thermal properties becomes crucial. Leaf thermal properties are the characteristics related to heat exchange of a leaf with its surroundings, which determine the energy flux (i.e. radiation absorptance and emissivity) and storage (i.e. mass, thickness, and specific heat capacity; [3]).These properties interact with microclimatic variables, influencing the energy balance and the rate of change of leaf temperature [30]. Furthermore, they play a role in regulating energy fluxes between plants and their environment, thereby linking environmental changes to plant functionality [3]. However, the variation of leaf thermal properties in response to environmental fluctuations remains inadequately established, largely due to the difficulty in accurately estimating them.

Under steady-state conditions, the energy balance of the leaf only requires consideration of the interactions among energy fluxes: net radiation, latent heat, and sensible heat fluxes. However, under a non-steady environment, a parameter (k) that is comprised of the product of leaf thickness, leaf density (on a fresh mass basis) and specific heat capacity, which describes the energy storage of the leaf, plays an important role by decoupling leaf temperature variations from the ambient temperature [30, 38]. Further, when applying the leaf energy balance equation, variations in the value of k also affect the estimation of other parameters. For example, for every 1% change in k, the amount of absorbed irradiance as estimated from thermal images is affected by 1% [44]. However, most studies only use rough estimates of leaf thermal properties, especially specific heat capacity (C_p_), which may affect the accuracy of thermal imaging applications under dynamic environments. C_p_ refers to the amount of heat absorbed or released per unit mass of a substance when the temperature per unit mass changes, mainly affecting the heat transfer rate, and has been reported to be in the range of 3500–4000 J kg^−1^ K^−1^ in plant leaves [19]. Plants with higher C_p_ may have a greater heat tolerance by dampening temperature variations and keeping leaf temperature closer to metabolic optima, while lower C_p_ may provide stronger atmospheric coupling and heat dissipation [22, 35, 36]. Thus, C_p_ is an essential trait for comprehending and forecasting the impacts of energy exchange on vegetation. However, an accurate estimation of C_p_ has been held back by a lack of convenient methods.

Traditionally, C_p_ was measured using differential scanning calorimetry, which is based on measuring the heat released or absorbed by a material during temperature changes, and relies on simple mixture rules [11]. A leaf can be considered as a mixture of water and various dry matter components, and its C_p_ can be determined depending on the C_p_ of water and dry mass depending on their proportions [30]. However, the accuracy of this method is constrained by its dependence on small masses of low-density biomass samples (low signal-to-noise ratio), and is destructive [5, 11]. Another commonly used method is the photoelectric technique, in which a material placed on the detector is irradiated with modulated light and the thermal properties are measured by detecting the phase and amplitude of the generated thermal waves [16]. However, this technique takes tens of minutes of measuring time per sample, during which g_sw_ and thus E may change, which would affect the resulting value. In recent years, some non-destructive and contactless techniques for C_p_ measurement have been developed, such as laser probing [6]: in a leaf sample exposed to a short laser pulse, C_p_ can be derived from the energy obtained from laser heating, divided by the product of the leaf temperature increase and its physical properties (heated leaf volume and leaf density). However, setting up the laser and determining its power and spot area are non-trivial tasks that, along with sensitivity to fluctuating environments, pose challenges for the wider application of this method. Furthermore, measuring only one sample at a time limits the applicability of this method to large-scale sample analysis. Therefore, a convenient and easy-to-use method for C_p_ measurements is highly desirable.

We have previously developed a method to derive the irradiance absorbed by leaves in whole plants, by fitting a leaf energy balance model to transient changes in leaf temperature [44]. Building on this work, we aimed to design a non-destructive and accurate method to quantify C_p_, by applying a light pulse (10 s) with known absorbed irradiance and leaf properties (density × thickness) in a controlled environment. The application of thermography allowed for contactless and rapid detection of C_p_, for the first time allowing the high-throughput estimation of C_p_. Additionally to developing the method, we estimated C_p_ in 13 plant species, thereby generating a new set of reference values for future applications of thermography in dynamic environments.

## Material and methods

### Theory

When exposed to a brief (~ 10 s) and intense light pulse (several hundred W m^−2^), a leaf undergoes rapid temperature elevation. The rise in temperature is mainly influenced by absorbed irradiance (short- and longwave radiation) and k (k = leaf thickness (lt) * leaf density (ρ) *specific heat capacity (C_p_)). Among the properties affecting k, C_p_ affects the response rate of leaf temperature (T_leaf_; [4]). When the light pulse stops, T_leaf_ follows an exponential decline until its value stabilizes at a lower value than air temperature (T_air_) due to evaporative cooling, and the rate of this is mainly affected by boundary layer conductance (including: heat transfer conductance, g_bh_ and water vapour conductance, g_bw_) and stomatal conductance (g_sw_). With measured absorbed irradiance and leaf characteristics (thickness and density), a leaf energy balance model can be fitted to transient changes of T_leaf_ during and after the exposure to a light pulse, by adjusting the values of k, g_sw_ and g_bh_ using Bayesian inference (see below for more details). Values minimizing the difference between modelled and observed T_leaf_ can be selected, and C_p_ can be determined by dividing k by the product of thickness and density. Utilizing the dynamic leaf energy balance model, T_leaf_ kinetics can be calculated [44]:

1 $$\frac{{{\text{dT}}_{{{\text{leaf}}}} }}{{{\text{dt}}}} = \frac{{\alpha {\text{I}}_{{\text{s}}} + 2\varepsilon \theta \left( {{\text{T}}_{{{\text{reflect}}}}^{4} - {\text{T}}_{{{\text{leaf}}}}^{4} } \right) - 2\rho_{air} {\text{C}}_{{\text{s}}} {\text{g}}_{{{\text{bh}}}} \left( {{\text{T}}_{{{\text{leaf}}}} - {\text{T}}_{{{\text{air}}}} } \right) - 2\lambda {*}\frac{{0.622{*}\rho_{air} }}{{P_{atm} }}{*}\left( {1/\left( {1/{\text{g}}_{{{\text{sw}}}} + 1/{\text{g}}_{{{\text{bw}}}} } \right)} \right){\text{*VPD}}}}{k}$$

where the term $\frac{d{T}_{leaf}}{dt}$ represents the rate of leaf temperature (K) change over time, which can be estimated by using a smooth spline that is fit to the observed temperature data and then is used to calculate the derivative of the fitted function at any time t (s) [38]. Other elements of Eq. 1 include α, the leaf short-wave radiation absorptance(dimensionless ratio of absorbed to incident radiation); I_s_, the short-wave radiation (W m^−2^); ℇ, the long-wave radiation emissivity (Table 1); θ, the Stefan-Boltzmann constant (W m^–2^ K^–4^); T_reflect_, the long-wave radiation that is reflected to the thermal camera by a crumpled piece of aluminum foil near the leaf (which is assumed to equal the temperature of the leaf’s surroundings; the term θT^4^_reflect_ thus represents all longwave energy received from the leaf’s environment); T_air_, the air temperature (K); ρ_air_, the air density (kg m^–3^); C_s_, the specific heat capacity of humid air (J kg^–1^ K^–1^); g_bh_, the boundary layer conductance to heat transfer (m s^−1^); λ, the latent heat of evaporation of water (J kg^–1^); $\frac{0.622\uprho }{{\text{P}}_{\text{atm}}}$, a conversion factor from P_a_ to kg m^–3^; P_atm_, the air vapour pressure (P_a_). g_sw_, the stomatal conductance to water vapour (m s^–1^), g_bw_, the boundary layer conductance to water vapour transfer (m s^–1^). Note that for consistency, g_sw_ and g_bw_ used the same unit as g_bh_ (m s^−1^) and can be converted to molar units (mol m^−2^ s^−1^), by using the conversion factor $\frac{\text{R}{T}_{leaf}}{{\text{P}}_{\text{atm}}}$ (R, the gas constant: 8.3145 J mol^−1^ K^−1^). In a typical laminar flow over plant leaves, g_bh_ ≌ 0.92 g_bw_ [18]. VPD, the difference between leaf internal vapour pressure (es) and air vapour pressure (ea; Pa; where ea is related to the air relative humidity (RH_air_) and T_air_, while es is a function of T_leaf_). ρ is the leaf density (kg m^–3^); *C*_*p*_, the specific heat capacity of the leaf (J kg^–1^ K^–1^); and lt, the leaf thickness (m). k represents the energy per unit area required to change the temperature of the material by 1 °K (J m^–2^ K^–1^). For consistency, for g_sw_ the same unit as for g_bh_ was used (m s^−1^); this can be converted to molar units using the conversion factor $\frac{0.92{\text{RT}}_{\text{leaf}}}{{\text{P}}_{\text{atm}}}$(mol m^−2^ s^−1^; with R the gas constant: 8.3145 J mol^−1^ K^−1^). Please note that g_bh_ and g_sw_ were considered constant during the light protocol and estimated using Bayesian inference (see below) as described by [44]. In this previous study, parameter estimations were validated against observations using lysimetric methods.

**Table 1:** Key measured leaf traits in 13 plant species

Common name	Latin name	Fresh weight (g)	Dry weight (g)	Leaf area (cm^2^)	Water loss (%)	Long-wave radiation emissivity	Short-wave radiation absorptance
avocado	*Persea americana*	1.359 ± 0.07	0.533 ± 0.03	32.404 ± 1.1	2.499 ± 0.5	0.960 ± 0.002	0.949 ± 0.003
broad bean	*Vicia faba*	0.446 ± 0.01	0.052 ± 0.002	18.846 ± 0.3	1.151 ± 0.3	0.993 ± 0.001	0.875 ± 0.005
coffee	*Coffea arabica*	1.606 ± 0.07	0.523 ± 0.03	71.727 ± 2.3	1.159 ± 0.2	0.973 ± 0.007	0.948 ± 0.003
cucumber	*Cucumis sativus*	2.346 ± 0.26	0.329 ± 0.04	110.875 ± 11.3	n.d	0.980 ± 0.005	0.919 ± 0.008
grapefruit	*Citrus paradisi*	1.455 ± 0.08	0.50 ± 0.04	52.914 ± 2.4	1.019 ± 0.2	0.948 ± 0.002	0.952 ± 0.002
orange	*Citrus sinensis*	1.294 ± 0.09	0.555 ± 0.05	46.005 ± 2.1	1.602 ± 0.4	0.947 ± 0.001	0.952 ± 0.003
Phalaenopsis	*Phalaenopsis amabilis*	6.304 ± 1.05	0.423 ± 0.07	48.923 ± 6.1	0.107 ± 0.04	0.909 ± 0.007	0.90 ± 0.005
pumpkin	*Cucurbita maxima*	3.050 ± 0.23	n.a	146.305 ± 10.7	2.902 ± 0.5	0.993 ± 0.004	0.887 ± 0.01
strawberry guava	*Psidium cattleianum*	0.824 ± 0.05	0.314 ± 0.03	21.312 ± 0.8	0.526 ± 0.01	0.960 ± 0.001	0.948 ± 0.002
sugar bean	*Pisum sativum*	0.203 ± 0.01	0.030 ± 0.002	10.447 ± 0.4	1.161 ± 0.3	0.985 ± 0.003	0.910 ± 0.007
sweet pepper	*Capsicum annuum*	0.921 ± 0.04	0.122 ± 0.005	40.019 ± 1.4	1.309 ± 0.1	0.997 ± 0.001	0.928 ± 0.002
tomato	*Solanum lycopersicum*	0.428 ± 0.03	0.049 ± 0.003	17.348 ± 1.1	3.637 ± 0.2	0.986 ± 0.002	0.901 ± 0.003
weeping fig	*Ficus benjamina*	0.501 ± 0.03	0.149 ± 0.01	24.123 ± 0.8	0.473 ± 0.01	0.939 ± 0.003	0.931 ± 0.002

Data represent means ± SE (n = 8). n.d—not determined, as petioles were placed in water-filled tubes upon detachment from the stem; n.a—not available due to data loss. Species are listed in alphabetical order.

Equation 1 utilized three input variables (T_air_, RH_air_, and T_reflect_), as well as three parameters deduced using Bayesian inference (g_bh_, g_sw,_ and k), to predict the temporal kinetics in T_leaf_ during a short light pulse. Other measurable parameters (α, I_s_, and ℇ) were determined independently (see Measurement protocols). lt can be calculated from the ratio of leaf volume to leaf area, and ρ can be calculated from the ratio of leaf fresh weight to leaf volume [40]. Therefore, the lumped term ρ * lt (kg m^−2^) can by calculated by dividing the leaf fresh weight by the leaf area. It is worth emphasizing that leaf dry weight divided by leaf area is often used to represent leaf mass per unit area (LMA) [17]. However, the use of fresh weight-based rather than dry weight-based calculations may be a better reflection of the variation in leaf volume and water content, and has previously been closely related to C_p_ [1]. Furthermore, k was first deduced by Bayesian inference, after which C_p_ can be obtained by dividing k by ρ * lt.

### Bayesian inference

The energy balance equation (Eq. 1) underwent parameterization through Bayesian inference, by adjusting simultaneously the values of g_bh_, g_sw_, and k. In short, the procedure automatically sampled several thousands of g_bh_, g_sw_, and k values that maximized the likelihood between the predictions and observations and using an algorithm that guide the sampling to explore the parameter space draw a probability distribution per parameter. A 95% Bayesian credible interval was computed per parameter, thereby quantifying the uncertainty of parameter estimation. Additionally, the credible interval for k derived from Bayesian inference already includes the impact of uncertainty and correlation with g_sw_ and g_bh._ The procedure was implemented using Stan (version 2.33.1; http://mc-stan.org/) with the cmdstan R interface, involving four MCMC runs (1000 iterations per chain, 500 for warm-up) ensuring an effective sample size > 200. Chains were validated using the diagnose function, confirming reliability [7]. Prior distributions representing our prior knowledge about the parameter values were broadly chosen (mean: 0, 0; sd: 0.005, 0.005 for g_sw_ and g_bh_ respectively). Model accuracy was evaluated using root mean squared error (RMSE). The above procedure was done separately per biological replicate, each of which was selected from different but uniformly growing plants of the same species.

### Plant material

Seeds of important horticultural crops, namely sweet pepper (*Capsicum annuum*), tomato (*Solanum lycopersicum*), cucumber (*Cucumis sativus*), pumpkin (*Cucurbita maxima*), broad beans (*Vicia faba*), and sugar beans (*Pisum sativum*) were sown into moist stone wool plugs in a growth chamber. Once germinated, plants were transplanted into stone wool cubes (10 × 10 × 7 cm) in a controlled-environment climate chamber and irrigated twice daily (7:00 am and 19:00 pm) with a nutrient solution [20] via an automatic ebb-and-flow system. The photoperiod was 16 h, with temperature setpoints of 23/20 °C (day/night), ambient CO_2_ levels of ~ 450 ppm, and relative humidity at 70 ± 2%. Plants grew under a dynamically changing irradiance, following a sinusoidal light pattern resembling natural conditions [44]. The average photosynthetic photon flux density (PPFD; 400–700 nm) during the photoperiod was 250 µmol m^−2^ s^−1^, with a maximum of 320 µmol m^−2^ s^−1^ and a minimum of 120 µmol m^−2^ s^−1^ Daily light integral (DLI) was 14.4 mol m^−2^ d^−1^ (Additional file 1: Fig. S1). After around 3–4 weeks, upper fully-expanded leaves were chosen for measurements.

Another six species, namely orange (*Citrus sinensis*), grapefruit (*Citrus paradisi*), coffee (*Coffea arabica*), avocado (*Persea americana*), strawberry guava (*Psidium cattleianum*), and weeping fig (*Ficus benjamina*; Additional file 2: Fig. S2) were grown in soil in two greenhouse compartments at Wageningen University, the Netherlands (52°N, 5.5°E). All plants were irrigated with tap water by hand once or twice a week, as needed. The soil was regularly fertilized with cow manure and tree bark. During the measurement period (April-June 2023), average day/night air temperatures and relative humidities were 22.4/18.3 °C and 60.4/68.5%, respectively. A shade screen (Ludvig Svensson, Sweden) was closed when solar radiation outside the greenhouse exceeded 500 W m^−2^. DLI in the greenhouse compartment was approximately 15–25 mol m^−2^ d^−1^. In addition, potted Phalaenopsis orchids *(Phalaenopsis amabilis)* were purchased from a local garden center (Tuincentrum De Oude Tol, Wageningen, the Netherlands). To minimize the effects of plant age and sampling location, healthy, clean, and similar sized leaves were selected in all cases.

### Measurement setup

**Fig. 1:**
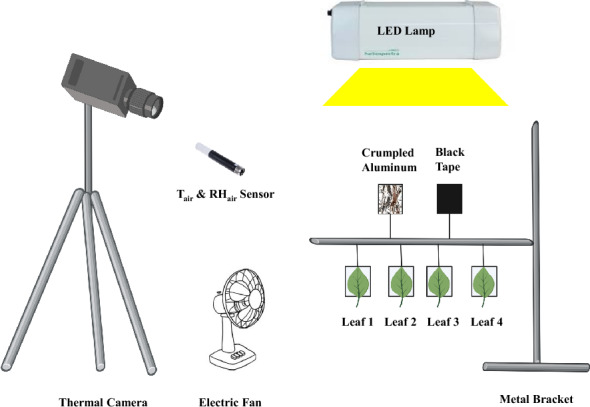
Schematic diagram of the experimental setup in the enclosure. From left to right: a thermal camera was used to capture object temperature, an electric fan to ensure full air mixing, a sensor to measure air temperature and relative humidity, and an LED lamp to provide a light pulse. The metal bracket on the right has six metal frames that hold four test leaf samples, a crumpled piece of aluminum foil (to estimate long-wave radiation emitted by the surroundings), and a piece of black tape (to determine light turn-on time). Figure 1 was created with BioRender.com

Measurements were conducted in a light-proof enclosure (210 L × 120 W × 75 H cm) made of metal, with wood panels at the top and bottom in a temperature-controlled laboratory with T_air_ of 20 °C. The enclosure was fully covered with black fabric to maintain a dark and stable environment. Inside the enclosure, a thermal camera (FLIR A655sc; FLIR system, Inc., Wilsonville, OR, USA) with an uncooled microbolometer detector (resolution: 640 × 480 pixels; spectral range: 7.5–14.0 µm; noise equivalent temperature difference: < 30 mK) was mounted on a tripod and placed on the left side of the enclosure. T_leaf_ was continuously captured using the ResearchIR max software (FLIR, version 4.40.12.38) of the camera. A LED light source (Elixia; Heliospectra AB, Göteborg, Sweden; containing a blue (peak: 450 nm), red (660 nm) and white LED channel, 44% blue, 11% green, 44% red and 0% far-red), located on the right side of the enclosure, was used to provide illumination. Between lamp and thermal camera, an electrical fan was placed to ensure good air mixing, increase g_bh_ and reduce temperature gradients. T_air_ and RH_air_ were recorded every 5 s by a sensor (HC2A-WIN-USB; Rotronic instruments, Crawley, UK) (Fig. 1).

A horizontal metal rod was placed 50 cm from the lamp and connected to the support frame. On one side of the rod, there were four small frames (5.5 L × 5.0 W cm) made of metal wires, which were used to hold leaf samples. On the other side of the rod, another two metal frames of identical dimensions were placed, one of which held a piece of crumpled aluminum foil, while the other was covered with black vinyl electrical tape (Additional file 3: Fig. S3). The temperature of the aluminum foil, when the longwave emissivity was set to 1, represents T_reflect_, providing an estimation of longwave radiation emitted by the surrounding environment [25]. Our previous work confirmed that the longwave radiation estimated with a small aluminum foil was representative of the entire measurement region [44]. The temperature response time of the black tape was used to determine when the light was turned on, while a K-type thermocouple was inserted into the black tape (ε = 0.97) to further verify the temperature accuracy captured by the camera. PPFD and irradiance (400–700 nm, W m^−2^ were measured in four locations per metal frame with an LI-180 spectrometer (LI-COR, Lincoln, NE, USA, and an average value (n = 4 per metal frame was used in later analyses. Using an anemometer (Voltcraft PL-135 HAN; Conrad Electronics, Hirschau, Germany, wind speed was measured and found to be consistent across all metal frames (0.20–0.22 m s^−1^ in all cases), indicating uniform air mixing and similar boundary layer conductance (given similar dimensions).

### Measurement protocols

The entire measurement process lasted ~ 10 min per leaf sample, including sample preparation and handling (3 min), thermal imaging protocol (2 min), and measurements of several leaf properties (leaf area, leaf light absorptance and long-wave radiation emissivity; altogether 5 min).

#### Preparations

Plants cultivated in the growth chamber were moved to the set-up for overnight dark adaptation prior to measurements. In the case that plants were cultivated in the greenhouse-, leaves were quickly cut from stems, wrapped in wet paper towels, placed in airtight plastic bags with high CO_2_ to close the stomata (~ 10 min), and brought to the lab. Then, excess water was wiped off leaf surfaces, and fresh weight (FW) was determined using a precision scale (precision of 0.01 g; Thermo Fisher Scientific Inc., Leicestershire, UK). For species prone to rapid water loss such as cucumber, leaf petioles were immediately immersed in a water-filled tube upon detachment from the stem.

#### Thermal imaging protocol

Four leaf samples were placed on metal frames and then exposed to a 10 s light pulse of 1250 µmol m^−2^ s^−1^ PPFD (irradiance: 270 W m^−2^), followed by a dark period lasting 110 s. The entire light protocol lasted 2 min. The rate of temperature increase (^o^C s^−1^) during light pulse exposure was calculated by the maximum temperature difference during the light pulse divided by exposure time (10 s). The lens of the thermal camera was positioned at a 45° angle and 60 cm above the leaf samples, and 25 thermograms were captured per second (25 Hz). Then, leaves were immediately weighed again to calculate the amount of water lost during thermal imaging. Water loss (the ratio of the difference in fresh weight before and after imaging protocol to the initial fresh weight) was < 4% across species (Table 1). Per species, eight leaves were measured (n = 8).

#### Leaf area, light absorptance, specific leaf area, and leaf water content

After thermal imaging, leaf samples were placed on a white board with a ruler for photographing. ImageJ open-source software (version 1.53, 64-bit) was used for calculating leaf area from the images. Subsequently, leaf light absorptance (400–700 nm) was measured using a spectrophotometer connected to an integrating sphere on three biological samples per species [41]. Leaf dry weight (DW) was measured by placing leaf samples in an oven at 80 ℃ for 48 h until the weight remained constant; this was assessed by comparing dry weights at 47 h and 48 h. Please note that the DW of pumpkin leaves was not recorded due to the accidental loss of data. Specific leaf area (SLA; m^2^ kg^−1^) was estimated on a fresh weight (SLA_FW_) basis to reflect leaf thickness, whereas LMA (g m^−2^) was calculated by dividing dry weight by leaf area. Leaf water content (LWC) was calculated by the following formula:

2 $$LWC\left(\%\right)=\frac{FW(g)-DW(g)}{FW(g)}*100$$

Leaf longwave radiation emissivity was determined by using the reference emittance technique proposed by [24]. Specifically, a beaker filled to 2/3 with water was heated to ~ 40 °C on a heating plate, and a magnet inside the beaker stirred the water (). The freshly cut leaf sample was floating in the water on a metal grid, to keep the leaf in position. A crumpled aluminum foil was placed next to the leaf to represent the longwave radiation of the surroundings. Air temperature and humidity were measured by a sensor (HC2A-WIN-USB; Rotronic instruments) placed next to the wall of the beaker, while four Chromel–Alumel thermocouple probes (K-type, Pico Technology Ltd., UK) were arranged at the same depths in the water to determine the uniformity of the water temperature. The lens of the thermal camera was placed at 40 cm, perpendicular to the water surface, to capture the water surface (T_water_), apparent leaf (T_l,app_), and aluminum foil temperatures (T_reflect_), respectively. This method assumed the water temperature to be equal to apparent leaf temperature. Leaf longwave radiation (${\upvarepsilon }_{\text{leaf}}$) emissivity was estimated as:

3 $${\upvarepsilon }_{\text{leaf}}={\upvarepsilon }_{\text{reference}}* \frac{{\text{T}}_{\text{l},\text{ app}}^{4}-{\text{T}}_{\text{reflect}}^{4}}{{\text{T}}_{\text{water}}^{4}-{\text{T}}_{\text{reflect}}^{4}}$$

where ε_reference_ is the emissivity of water (ε = 0.98, spectral range: 7.5–14.0 µm),T_l,app_ is the leaf temperature with ε_reference_. This procedure was repeated for four leaf samples per species (Table 1; Additional file 4: Fig. S4).

### Method validation

#### Determining the C_p_ of an aluminum plate

To validate our method, a material with known properties was subjected to the thermal imaging protocol described above. An aluminum plate painted black with a known thickness of 0.001335 m, density of 2484 kg m^−3^, specific heat capacity (C_p_) of 896 J kg^−1^ K^−1^ (https://gchem.cm.utexas.edu/index.php), emissivity (ε) of 0.96 and absorbance of (α) of 0.98 was used, resulting in a k of 2968 J m^−2^ K^−1^. This plate was placed on a metal frame and subjected to a 1 min light pulse of 1250 µmol m^−2^ s^−1^ PPFD (corresponding to irradiance of 270 W m^−2^) followed by 9 min of darkness. Due to the large value of k of aluminum, we prolonged the light exposure time compared to that used on leaf samples. The aluminum plate was placed on each of the four metal frames, and the process was thus repeated four times (four technical replicates; n = 4). The temperature of the aluminum plate was captured using the thermal camera. Equation 1 was used to derive C_p_, using known irradiance absorptance, emissivity, and physical properties of the black aluminum plate, as well as measured absorbed irradiance.

#### Sensitivity analysis

To characterize the extent to which changes in parameters in Eq. 1 affect the estimation of C_p_, a sensitivity analysis was performed. The effects of ± 10% changes in short-wave radiation absorptance, long-wave radiation emissivity, temperature of the aluminum foil (T_reflect_), irradiance, air temperature, relative humidity, and aluminum thickness on C_p_, using the dataset from the black aluminum plate placed on each of the four metal frames, were calculated.

#### Comparison between attached and detached leaves

C_p_ was assessed on sweet pepper and tomato leaves in an attached and a detached state (i.e., the measurement was repeated twice on the same leaf) to test whether detached leaves could be used for C_p_ determination. The experiment was conducted in twelve tomato and three sweet pepper plants), whose leaves were selected from the top of the canopy. Similarly sized leaves of uniformly growing plants were used.

### Data analysis

**Fig. 2:**
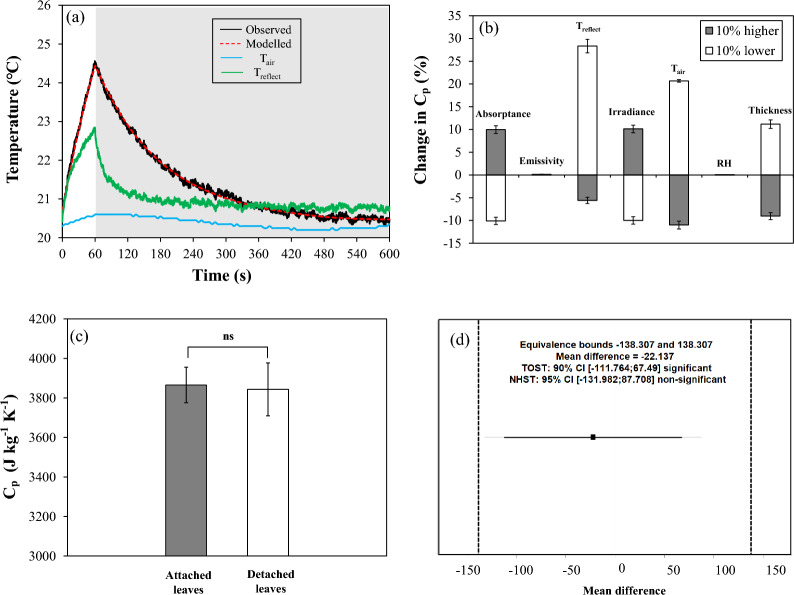
Testing of a thermography-based method to determine C_p_. **a** Validation of C_p_ based on temperature kinetics of a black aluminum plate under a 60 s light pulse (270 W m^−2^) followed by 9 min darkness. Solid black, blue, and green lines represent observed aluminum temperature, air temperature, and reflected temperature, respectively. The dashed red line represents modelled data of aluminum temperature kinetics, generated by fitting the leaf energy balance model to the observed temperature kinetics. The gray area represents darkness; **b** sensitivity analysis of C_p_ of the black aluminum plate when short-wave radiation absorptance, long-wave radiation emissivity, temperature of the aluminum foil (T_reflect_, K), irradiance (W m^−2^), air temperature (K), relative humidity (%), and aluminum thickness (m) were independently changed by + 10% (gray) and −10% (white). Bars show means ± SE (n = 4); **c** comparison of C_p_ in attached (i.e., on the stem) and detached (i.e., removed from the stem) tomato leaves. Bars show means ± SE (n = 12), ns indicates lack of significant statistical difference (*P* > 0.05); **d** Results of the TOST equivalence and null hypothesis significance tests (NHST) for the comparison of mean differences between attached and detached leaves. The black square represents the observed mean difference. The shorter horizontal line shows the TOST 90% confidence interval, while the longer horizontal line represents the NHST 95% confidence interval. Vertical dashed lines mark the equivalence bounds

Equation 1 and statistical analysis were implemented in R (R project, version 4.3.2; related code are available on https://github.com/jiayu0903/leaf-specific-heat-capacity-determination.git). When comparing datasets between attached and detached leaves, a Shapiro–Wilk test was employed to assess the normality of the data, while Levene's test was utilized to evaluate the homogeneity of variances. The dataset was compliant and subsequently a Student's t-test was utilized for paired samples to assess significant differences between means in Fig. 2c (P < 0.05). A two one-sided test (TOST) was conducted to assess equivalence using a paired t-test to determine if the mean difference between attached and detached leaves fell within predefined equivalence bounds. The 90% confidence interval (CI) was calculated for the equivalence test, while the 95% CI was used for the null hypothesis significance test (NHST) to evaluate the difference from zero.

## Results

### Validation of the energy balance model and experimental set-up

The temperature kinetics of the black aluminum plate under a 60 s light pulse first rose approximately linearly and then decreased exponentially until reaching a steady state (Fig. 2a). The model simulated the temperature kinetics of the black aluminum plate accurately (Fig. 2a; RMSE = 0.06 °C). Values of C_p_, derived from using the leaf energy balance model (Eq. 1), were 886 ± 13 J kg^−1^ K^−1^, and were thus very close to 890 J kg^−1^ K^−1^ as previously determined for aluminum. The sensitivity analysis further revealed that changes in long-wave radiation emissivity and relative air humidity caused negligible changes in C_p_, whereas T_reflect_ and T_air_ strongly affected C_p_ (Fig. 2b). Specifically, in- or decreasing T_reflect_ by 10% resulted in a 20–25% change in C_p_, while similar changes in radiation absorptance or leaf thickness changed C_p_ by roughly ± 10% (Fig. 2b). C_p_ values derived from either attached or detached leaves of tomato (Fig. 2c; *P* = 0.66) and sweet pepper (Additional file 5: Fig. S5; *P* = 0.25) were not significantly different from one another, suggesting that detaching leaves from the plant and keeping them wet before determining C_p_ was not problematic. The mean difference between detached and attached tomato leaves was only − 22 J kg^−1^ K^−1^ (Fig. 2d). This difference was well within the equivalence bounds (− 138, 138), which include TOST 90% and NHST 95% confidence intervals (Fig. 2d). This suggests that the difference between attached and detached leaves was small enough to be statistically insignificant, and C_p_ of attached and detached leaves can indeed be considered equivalent.

## C_p_ in several plant species, and relationships with other leaf properties

**Fig. 3:**
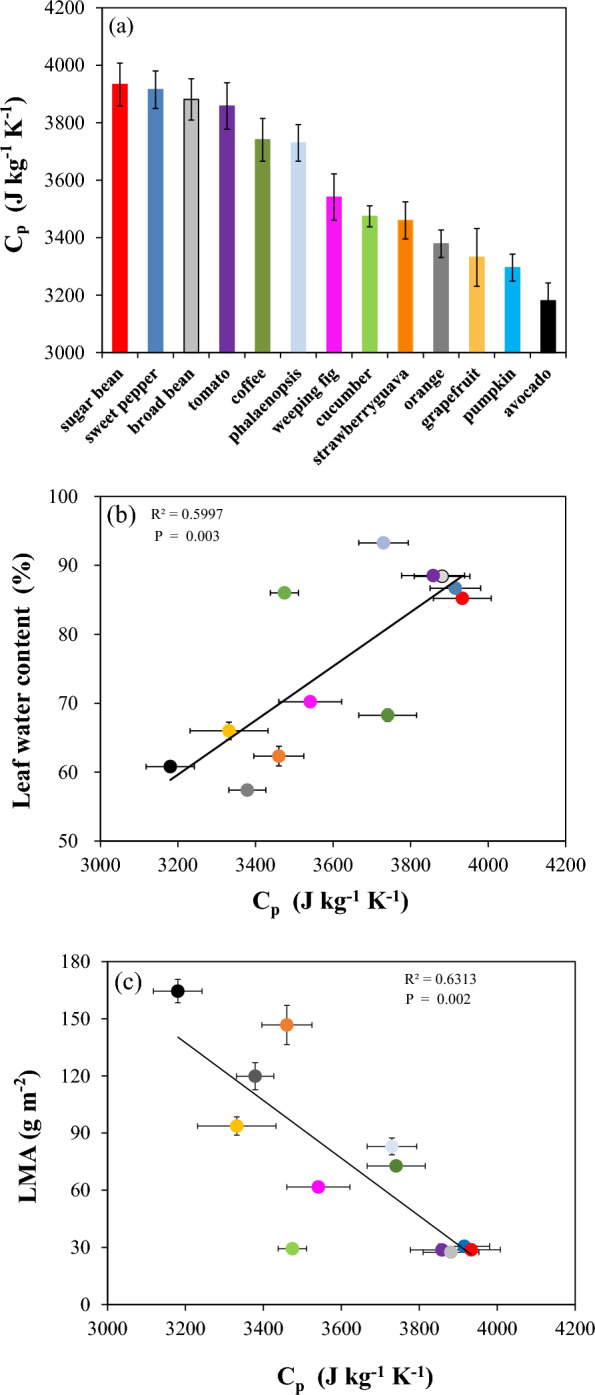
Species variation of C_p_ and its relationships with leaf morphology. **a** Specific heat capacity (C_p_) in 13 plant species. **b**, **c** Relationships between C_p_ and leaf water content (b, %) and leaf mass per area (c, LMA). The color of each dot indicates the species (colors as in Fig. 3a). Data shown in a-c represent means ± SE (n = 8). Due to data loss, data from pumpkin are not shown in Fig. 3b&c

Once our method was validated on aluminum (Fig. 2), we used it to determine C_p_ on a range of species (Fig. 3a). This was accomplished by including measured leaf properties in Eq. 1, such as short-wave absorptance (a), long-wave emissivity (ε), fresh weight & leaf area (SLA_FW_ = ρ * lt, kg m^−2^) (Table 1). C_p_ between species ranged from ~ 3185 (avocado) to ~ 3920 J kg^−1^ K^−1^ (sugar bean; Fig. 3a). There was a positive linear correlation between C_p_ and leaf water content (R^2^ = 0.60, *P* = 0.003), confirming once more that leaf water content was a key factor in determining C_p_ (Fig. 3b). Generally, there was a negative linear relationship (R^2^ = 0.63,* P* = 0.002) between C_p_ and LMA (Fig. 3c), and a similar trend was found between leaf water content and LMA (R^2^ = 0.65, *P* = 0.002, see Additional file6: Fig. S6). Thus, leaves with higher LMA, typically exhibited a lower leaf water content. Phalaenopsis did not follow the relationship between leaf water content and LMA (Fig. S6), likely due to its very thick and succulent leaves, allowing it to store large amounts of water to survive in environments with irregular water supply.

### Temperature changes across species and relationship with SLA

**Fig. 4:**
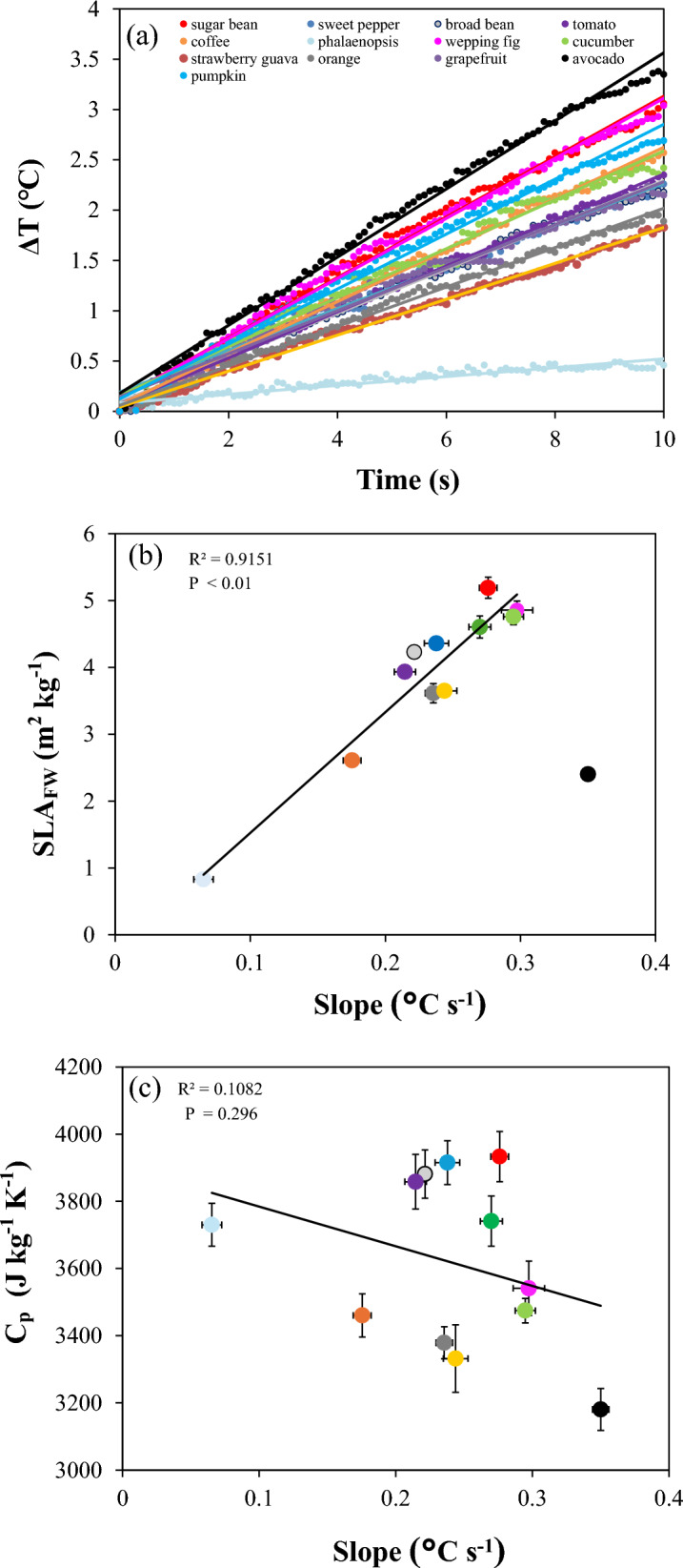
Relative temperature variation and its relationships with SLA_FW_. **a** Representative values of leaf temperature change during 10 s exposure to irradiance (270 W m^−2^) per species; the relationship between the slope of leaf temperature increase (^o^C s^−1^) during high irradiance exposure and **b** SLA_FW_ (m^2^ kg^−**1**^) and **c** C_p_ (J kg^−1^ K^−1^) (means ± SE, n = 8). Avocado was excluded from the linear relationship shown in (**b**). Due to data loss, data from pumpkin are not shown in Fig. 4b and c

The temperature increases during 10 s of high light irradiance (270 W m^−2^) exposure ranged from 0.46 °C to 3.38 °C among the 13 plant species (Fig. 4a). The steepest slope (°C s^−1^) in temperature change was found in avocado, which also happened to have the lowest C_p_ (Fig. 3a). Conversely, Phalaenopsis had the shallowest slope of temperature increase, which coincided with exceptionally thick leaves (lowest SLA _FW_; Fig. 4b) and high leaf water content (Fig. 3b). To conduct a full analysis of factors influencing k, we analyzed the correlation of C_p_, leaf water content, LMA, and SLA_FW_ with the slope of temperature rise during irradiance exposure. Surprisingly, C_p_, LMA, and water content did not greatly affect the temperature response (Fig. 4c; Additional file 7: Fig. S7), while SLA_FW_ correlated strongly and positively with it (Fig. 4b), suggesting that thinner leaves heated up more quickly.

## Discussion

### A new method to rapidly estimate specific heat capacity of plant leaves

We developed a rapid and accurate protocol, based on the leaf energy balance model, to estimate leaf specific heat capacity, and validated it using a material with known thermal properties (aluminum). We then used our new method to generate C_p_ values on a range of 13 horticultural and tropical species. Our method was initially validated by applying the leaf energy balance model to temperature transients before and after a light pulse on a black painted aluminum plate (Fig. 2a) and using Bayesian inference to derive g_sw_, g_bh_, and k values. Results closely matched values from an online database (https://gchem.cm.utexas.edu/data/section2.php?target=heat-capacities.php), affirming the accuracy of our approach. Further comparison of C_p_ determined on detached and attached leaves demonstrated that it was safe to apply the method to detached (dark acclimated) leaves (Fig. 2c), allowing us to apply the method to a broader range of plant species, including those from big trees. Traditional C_p_ measurements have often been destructive or involved cumbersome operational processes, thus complicating data acquisition and causing unnecessary water loss. This could potentially impact the final results, given the strong correlation between leaf water content and C_p_ (Fig. 3b), leading to inaccurate results. In contrast, our entire measurement protocol took only around 10 min, thus minimizing water loss and likely resulting in more accurate C_p_ results. Altogether, our method lends itself for screening large groups of plants for species variation in C_p_.

Average values of C_p_ in 13 horticultural crops as well as several tropical plant species were in the range of 3200—4000 J kg^−1^ K^−1^_,_ similar to the previously reported range of 3500—4000 J kg^−1^ K^−1^ [19]. Other published values for C_p_ include ~ 2252 J kg^−1^ K^−1^ in *N. benthamiana* [5]. Even lower values of around 1200–2300 J kg^−1^ K^−1^ were reported for leaves of tropical fruit crops [16], (Fig. 3a), and this difference to tropical species measured in our study could be attributed to differences in the methods and plant species used. The lower C_p_ in Jayalakshmy & Philip [16] may be the result of water loss during the relatively lengthy measurements (tens of minutes) used by the authors. In Buyel et al. [5], the low C_p_ in *N. benthamiana* likely stems from its adaptation to arid conditions, leading to lower leaf water content, and its thin, easily damaged leaves that may have lost considerable amounts of water during measurement.

Tropical and subtropical regions are at risk of extreme temperatures in the context of global warming (average temperature increase of 0.5 °C per decade), potentially impacting plant metabolism, reproduction, growth and survival [10]. Leaves with large C_p_ can dampen temperature fluctuations, helping them stay within the optimal range for photosynthesis for a larger fraction of time [22], in environments where rapid temperature changes could otherwise push leaf temperatures beyond the thresholds for effective photosynthesis. Leaves with lower C_p_ may be the result of acclimation and survival strategies, as tropical plants tend to have broader and thinner leaves (lower water content) that allow for rapid growth and light capture [12, 42]. Our approach provides an effective tool to screen for leaf C_p_; future studies may be able to connect it to other heat resistance mechanisms, including rapid accumulation of heat shock proteins and heat-stable Rubisco activase [15, 43].

Leaf C_p_ can be calculated based on leaf contents of dry mass and water with their respective heat capacity [22]. Indeed, our study also detected a strong positive relationship between C_p_ and leaf water content (Fig. 3b), consistent with previous research [1]. Water has a high specific heat capacity (4182 J kg^−1^ K^−1^,[19]), makes up a substantial portion of a leaf (~ 40–98% of leaf fresh mass for desiccated and turgid leaves, Pask et al., 2012), and therefore has significant effects on C_p_. However, it should be noted that the relationship between C_p_ and leaf water content ‘only’ had a R^2^ = 0.6, suggesting that C_p_ was determined by other factors as well. This also means that simply determining leaf water content– which would only require a scale and an oven – is insufficient to infer C_p_. It was previously stated that C_p_ also depends on fiber content and other organic components in the leaf [16], but their role in determining leaf C_p_ is not fully understood. It is noteworthy that LMA can be used as rough proxy of C_p_, although with quite some uncertainty (Fig. 3c; R^2^ = 0.63). LMA is widely used in trait studies [34], as it can be measured easily, but researchers have so far not connected this with leaf energy balance much. In summary, leaf C_p_ is a complex parameter influenced by a combination of factors, and our method provides a fast and accurate way to determine it.

In the leaf energy balance equation (Eq. 1), the rapidity of leaf temperature changes is also related to leaf traits, represented by the composite parameter k (thickness x density x C_p_ or C_p_ / SLA_FW_), but not each component contributes equally to k. Leaf thickness has a close relationship with heat transfer within the leaf. In general, excessively thick leaves may have longer heat transfer paths (i.e., routes through which heat travels in the leaf structure) and decrease the heat transfer efficiency and heating rate [2, 36]. SLA is a trait associated with plant resource use efficiency, impacting growth and ecological niches [13]. Fresh weight-based SLA (SLA_FW_) is a good indicator of fresh leaf thickness since it includes leaf water, making it more representative of leaf volume than LMA [40]. Our results revealed that SLA_FW_ correlated positively with the rate at which leaves heated up during high light exposure, while C_p_ did not. Thin leaves (high SLA_FW_) could have absorbed much more radiant heat per unit mass than thicker leaves, resulting in faster leaf heating. Although avocado has relatively thick leaves, it heated up fastest (Fig. 4b), mainly due to its lower C_p_ and leaf water content (Fig. 3a and b), which also represents low heat storage capacity and a rapid response to irradiance and ambient temperature fluctuations [30]. Additionally, the thick cuticle of avocado leaves somewhat prolongs the internal heat retention, leading to a sustained increase in temperature [31]. These results indicated that the factors affecting plant temperature response are species-specific. Previous studies suggested that C_p_ is important [1, 22] in leaf temperature response, however, our results suggested that the transient ‘temperature variations were primarily driven by SLA_FW,_ rather than C_p_ alone (Fig. 4b and c). This may be because SLA_FW_ is a composite parameter that encompasses multiple leaf traits— thickness, density, and water content — indicating that temperature variation arises from an interplay of correlated factors (thickness x density x C_p_) that cannot be considered in isolation. Similarly to LMA, SLA_FW_ is also an easily estimated parameter that can be used to infer the rapidity of leaf temperature change in the future.

### Potential limitations and future insight

While our method provides accurate C_p_ estimates, the sensitivity analysis highlighted its dependence on several key parameters. Changes of ± 10% in light intensity could lead to a ± 10% variability in C_p_ (Fig. 2b), meaning that light intensity and its spatial distribution should be characterized accurately, especially in species with larger leaves. By applying the method developed previously [44], we can obtain absorbed irradiance per pixel in the camera’s field of view, using thermography. T_air_ variation (−10% approx. −2.2℃) caused very large changes in C_p_ (+ 20%), emphasizing the need for a well-mixed and stable measurement environment and accuracy of T_air_ data. It was noted that a −10% change in T_reflect_ (approx. −2.1 ℃) resulted in a major increase of + 25% in C_p_; however, our previous experiments confirmed that the distribution of T_reflect_ is homogeneous under the same setup, and the difference in T_reflect_ measured at different locations was within ± 0.5 °C [44], thus this parameter is unlikely to lead to erroneous C_p_ estimates. To mitigate the potential bias introduced by noisy data, it is crucial to either wait long enough or to use a high irradiance so that the temperature rise of the leaf under irradiance exposure is large enough to differentiate it from random noise around a constant signal. For example, the typical noise of a thermocouple is ~ 0.2 ℃ [26], and therefore it is desirable to obtain a > 2 ℃ increase in leaf temperature for a sufficiently large signal/noise ratio. Also, the duration of light exposure may need to be adjusted based on species. In Phalaenopsis, leaf temperature only increased by 0.38 ℃ (Fig. 4a; in this case, a longer light exposure based on the rate of temperature increase would have likely increased the signal/noise ratio. For high-throughput purposes, we did not measure specific values of leaf density and thickness, but follow-up studies could involve these traits and explore their relationship with C_p_ and temperature response.

Our research further revealed that variations in leaf thermal properties, such as C_p_ and leaf thickness, may cause potential errors when applying thermography in unstable environments. A typical application of thermography is the monitoring of plant status under drought conditions in the field [8, 23]. Plants under drought conditions often experience a reduction in leaf water content [19], significantly impacting C_p_ (Fig. 3b), along with a change in leaf thickness [17, 21, 27]. These plant thermal properties, which vary with external environmental changes, collectively contribute to changes in k, a key trait of leaf energy balance (Eq. 1). Specifically, using a thermal imaging camera and considering the leaf energy balance to estimate physiological indicators that reflect plant growth status under different stress conditions, such as transpiration rate or stomatal conductance, will yield more accurate measurements if variations in k are quantified rather than ignored. Measuring leaf thermal properties separately is inefficient and labor-intensive, whereas adopting our method allows for direct quantification of k, which helps pave the way for more precise thermal imaging applications.

## Conclusions

Our study presents a rapid and easy thermography-based method for estimating leaf specific heat capacity. We validated the method with a black aluminum plate, and determined C_p_ values for 13 plant species, providing reference C_p_ values for future research. Our method can serve as a useful tool to improve the accuracy of thermography-based plant phenotyping and monitoring, as well as for improving predictions of plant-environment interactions in the face of global climate change. Our approach can also be used by plant breeders to select for C_p_, which may add to the toolbox of climate change mitigation in crops.

## Supplementary Information

Additional file 1: Figure S1. Daily irradiance pattern in the climate chamber

Additional file 2: Figure S2. Overview of tropical crops used in this experiment

Additional file 3: Figure S3. Picture of experimental setup

Additional file 4: Figure S4. Overview of the long-wave radiation emissivity measurement setup

Additional file 5: Figure S5. Comparison of C_p_ in attached and detached sweet pepper leaves.

Additional file 6: Figure S6. Relationship between leaf water content, and LMA.

Additional file 7: Figure S7. Relationship between slope of temperature increase in Fig. 4a andLeaf water content, andLMA.

## Data Availability

Related codes are available on GitHub (https://github.com/jiayu0903/leaf-specific-heat-capacity-determination.git).

## Energy balance parameters

C_p_: Specific heat capacity of leaf (J kg^−^^1^ K^−^^1^)
C_s_: Specific heat capacity of humid air (J kg^−^^1^ K^−^^1^)
ℇ_leaf_, ℇ_reference_: Long-wave radiation emissivity of leaf and water (dimensionless)
g_bh_: Boundary layer conductance to heat transfer (m s^−^^1^)
g_bw_: Boundary layer conductance to water vapour (m s^−^^1^)
g_sw_: Stomatal conductance to water vapour (J kg^−^^1^ K^−^^1^)
k: Amount of the energy per unit area required to change the temperature of the material by 1 °K (J m^–2^ K^–1^)
l: Leaf thickness (m)
R: Gas constant (m^3^ Pa K^−1^ mol^−1^)
t: Time (s)
T_leaf_: Leaf temperature (K)
T_reflect_: Temperature of a crumpled piece of aluminum foil (K)
VPD: Leaf to air vapour pressure deficit (Pa)
α: Leaf absorptance within short-wave radiation (400–700 nm) (dimensionless)
Θ: The Stefan-Boltzmann constant (W m^–2^ K^–4^)
λ: Latent heat of evaporation of water (J kg^–1^)
ρ, ρ_air_: Density of leaf and air (kg m^−^^3^)

## Environmental variables

es, ea: Leaf internal (es) and air (ea) vapour pressure (Pa)
I_s_: Incident short-wave radiations (W m^−^^2^)
P_atm_: Atmospheric pressure (Pa)
PPFD: Photosynthetic photon flux density (µmol m^–2^ s^–1^)
RH_air_: Air relative humidity (dimensionless)
T_air_: Air temperature (K)

## Measurement indicators

DW: Dry weight (g)
FW: Fresh weight (g)
LMA: Leaf mass per area (g m^−^^2^)
LWC: Leaf water content (%)
SLA_FW_: Specific leaf area calculated by fresh weight (m^2^ kg^−^^1^)
ΔT: Leaf temperature change during 10 s exposure to irradiance (^o^C)
