# Supplementary material for: A simple new method to determine leaf specific heat capacity
Source: Plant Methods. 2025 Jan 24;21:6. doi: 10.1186/s13007-025-01326-3 (PMC11759430; doi:10.1186/s13007-025-01326-3)
Supplement: Supplementary file 1 — Additional file 1: Figure S1. Daily irradiance pattern in the climate chamber [file 13007_2025_1326_MOESM1_ESM.docx]

**Additional Files**

Plant Methods Additional Files
Article title: A simple new method to determine leaf specific heat capacity
Authors: Jiayu Zhang, Elias Kaiser, Hanyi Zhang, Leo F.M Marcelis, Silvere Vialet-Chabrand^*^


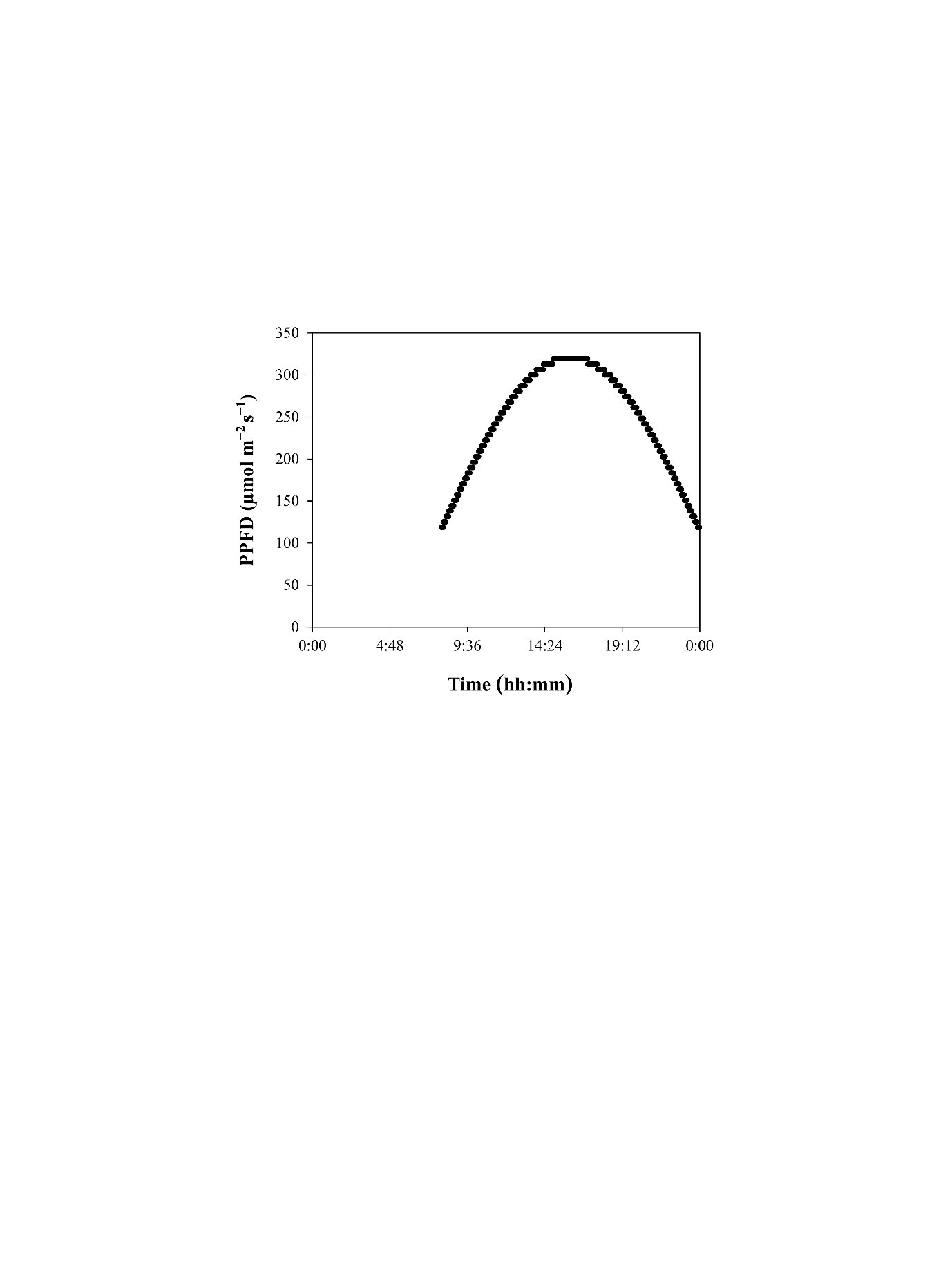


Figure S1. Daily irradiance pattern in the climate chamber.
