# Supplementary material for: A simple new method to determine leaf specific heat capacity
Source: Plant Methods. 2025 Jan 24;21:6. doi: 10.1186/s13007-025-01326-3 (PMC11759430; doi:10.1186/s13007-025-01326-3)
Supplement: Supplementary file 2 — Additional file 2: Figure S2. Overview of tropical crops used in this experiment [file 13007_2025_1326_MOESM2_ESM.docx]

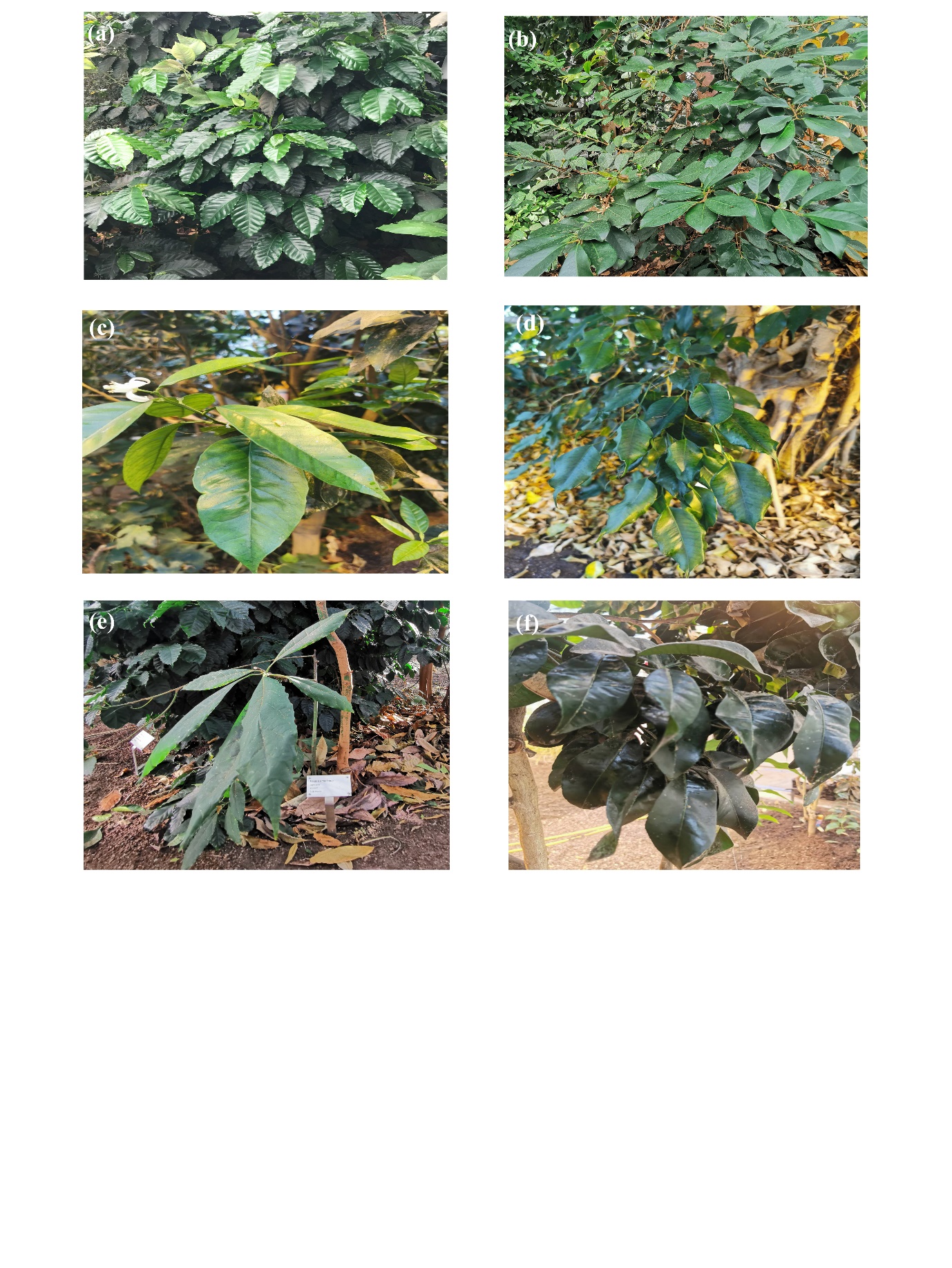


Figure S2. Pictures of tropical plant species in the greenhouse. (a) Coffee (*Coffea arabica*); (b) Strawberry guava (*Psidium cattleianum*); (c) Orange (*Citrus sinensis*); (d) Weeping fig (*Ficus benjamina*); (e) Avocado (*Persea americana*); (f) Grapefruit (*Citrus paradisi*).
