# Supplementary figures and images for: A simple new method to determine leaf specific heat capacity
Source: Plant Methods. 2025 Jan 24;21:6. doi: 10.1186/s13007-025-01326-3 (PMC11759430; doi:10.1186/s13007-025-01326-3)

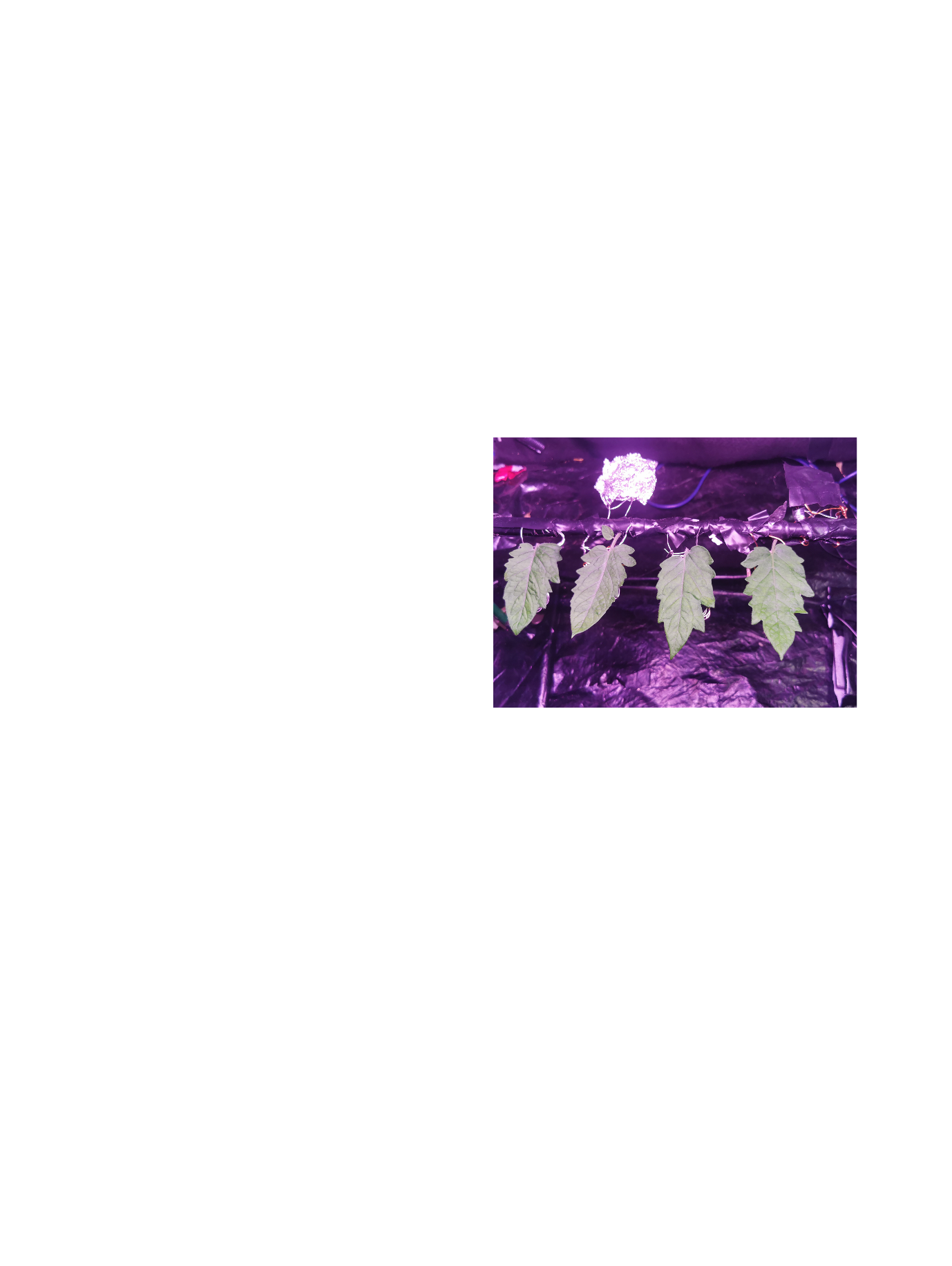


Figure S3. Picture the experimental setup (leaves of tomato, *Solanum lycopersicum*).

Supplement: Supplementary file 3 — Additional file 3: Figure S3. Picture of experimental setup [file 13007_2025_1326_MOESM3_ESM.docx]

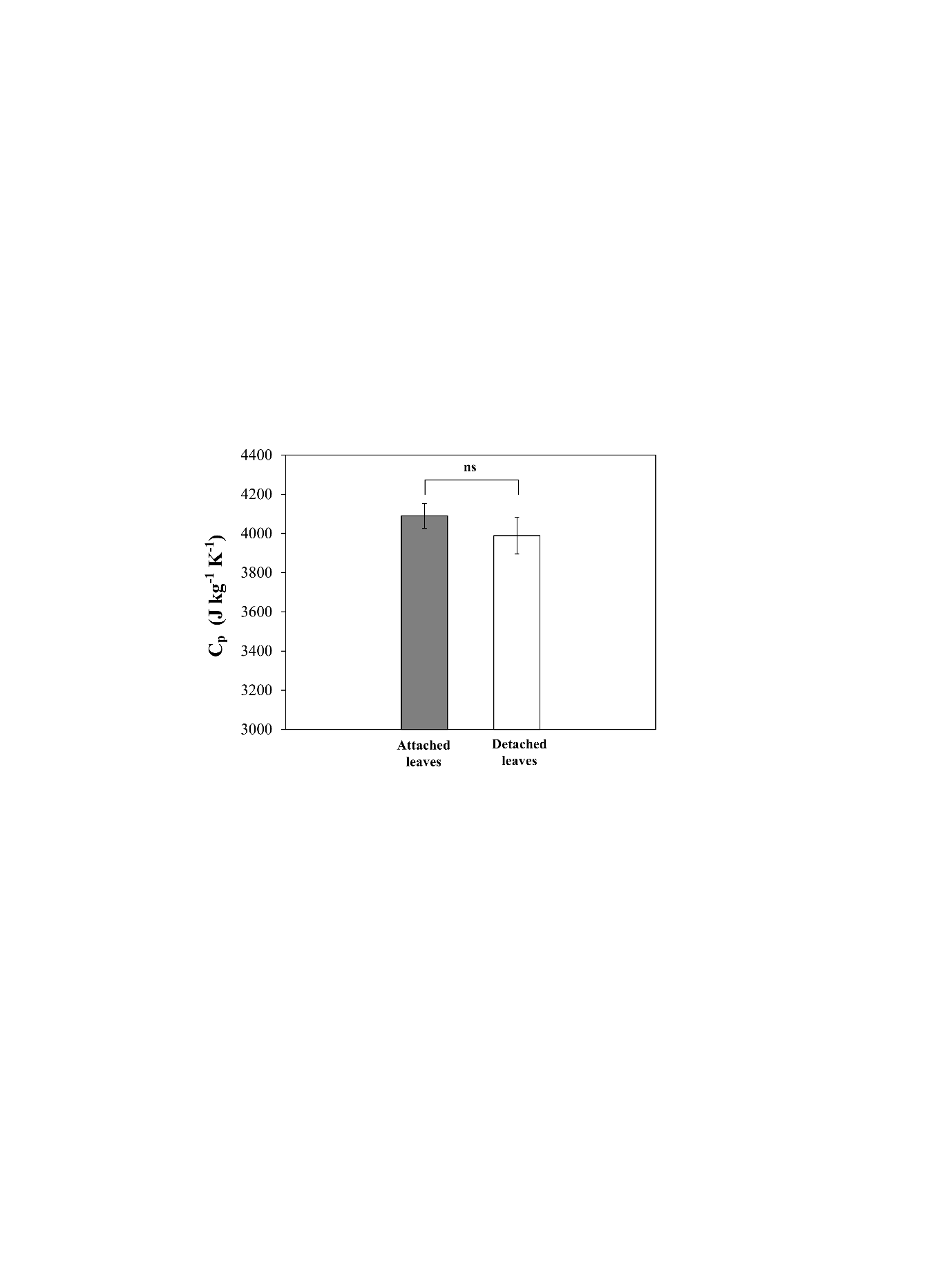


Figure S5. Comparison of Cp in attached and detached sweet pepper leaves.

Supplement: Supplementary file 5 — Additional file 5: Figure S5. Comparison of Cp in attached and detached sweet pepper leaves. [file 13007_2025_1326_MOESM5_ESM.docx]
