# Supplementary material for: A simple new method to determine leaf specific heat capacity
Source: Plant Methods. 2025 Jan 24;21:6. doi: 10.1186/s13007-025-01326-3 (PMC11759430; doi:10.1186/s13007-025-01326-3)
Supplement: Supplementary file 4 — Additional file 4: Figure S4. Overview of the long-wave radiation emissivity measurement setup [file 13007_2025_1326_MOESM4_ESM.docx]

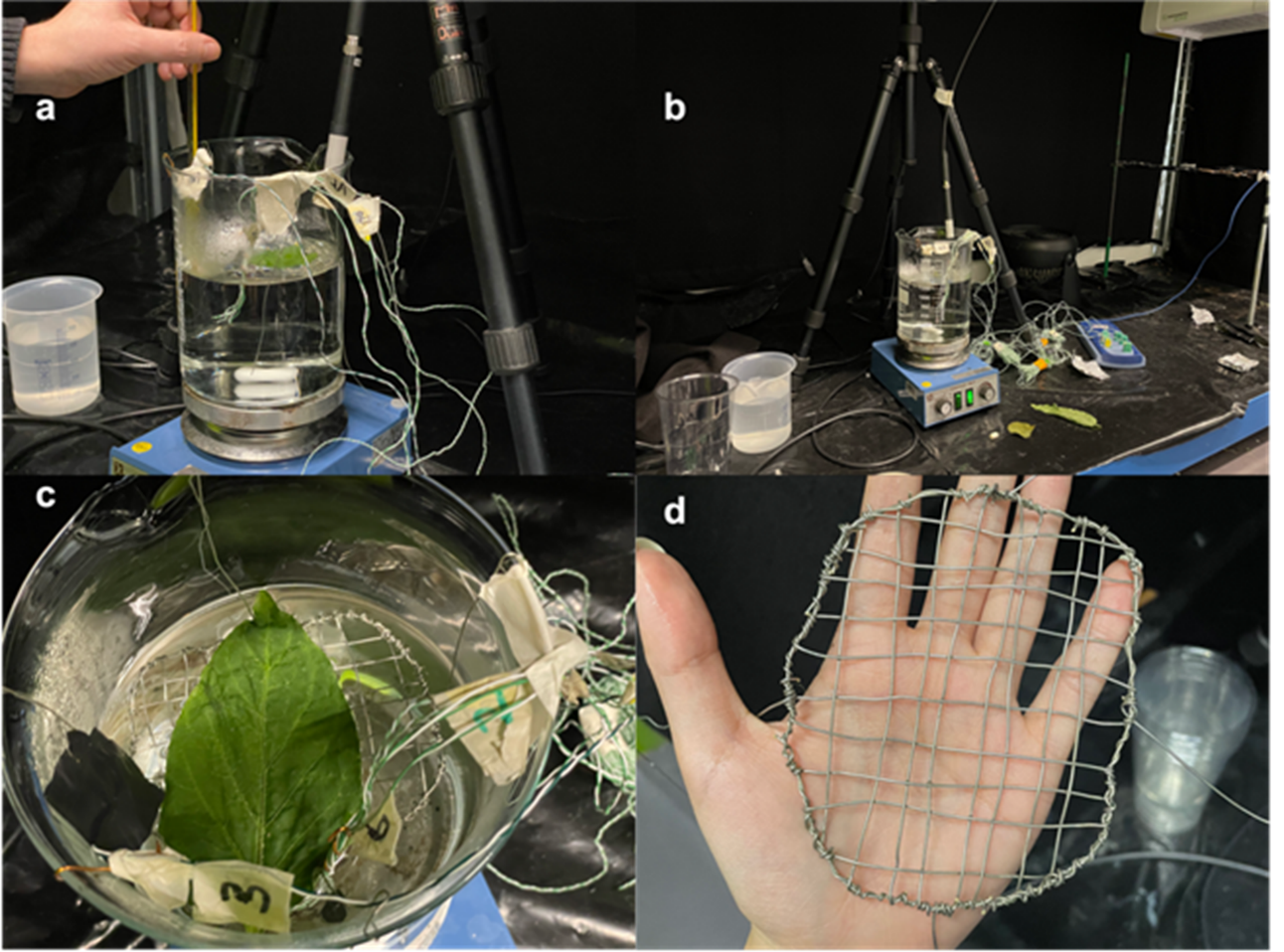


Figure S4. Overview of the long-wave radiation emissivity measurement setup. (a) Beaker on a heating plate, with thermocouples suspended in the water; (b) Location of heating plate and thermal imaging camera; (c) leaf of sweet pepper (*Capsicum annuum*) on a plate made of aluminum wire; (d) aluminum plate with four flexible cords secured to the edge of the beaker
