# Supplementary material for: A simple new method to determine leaf specific heat capacity
Source: Plant Methods. 2025 Jan 24;21:6. doi: 10.1186/s13007-025-01326-3 (PMC11759430; doi:10.1186/s13007-025-01326-3)
Supplement: Supplementary file 6 — Additional file 6: Figure S6. Relationship between leaf water content, and LMA. [file 13007_2025_1326_MOESM6_ESM.docx]

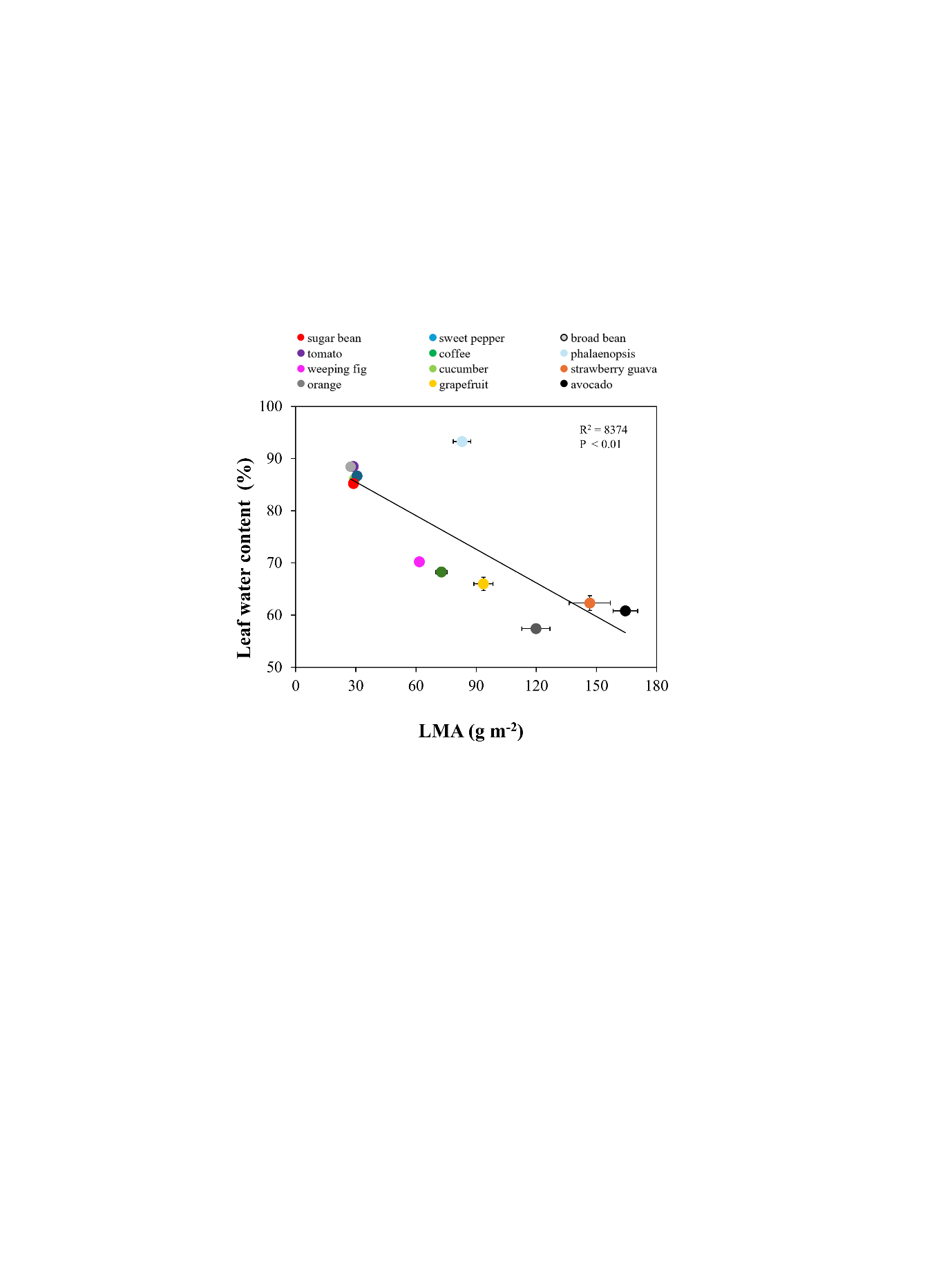


Figure S6. Relationship between leaf water content (%), and LMA (g m^-2^). The linear correlation does not include the data derived from phalaenopsis leaves (turquoise), and data derived from pumpkin leaves is not shown due to data loss.
