# Supplementary material for: A simple new method to determine leaf specific heat capacity
Source: Plant Methods. 2025 Jan 24;21:6. doi: 10.1186/s13007-025-01326-3 (PMC11759430; doi:10.1186/s13007-025-01326-3)
Supplement: Supplementary file 7 — Additional file 7: Figure S7. Relationship between slope of temperature increase in Fig. 4a andLeaf water content, andLMA. [file 13007_2025_1326_MOESM7_ESM.docx]

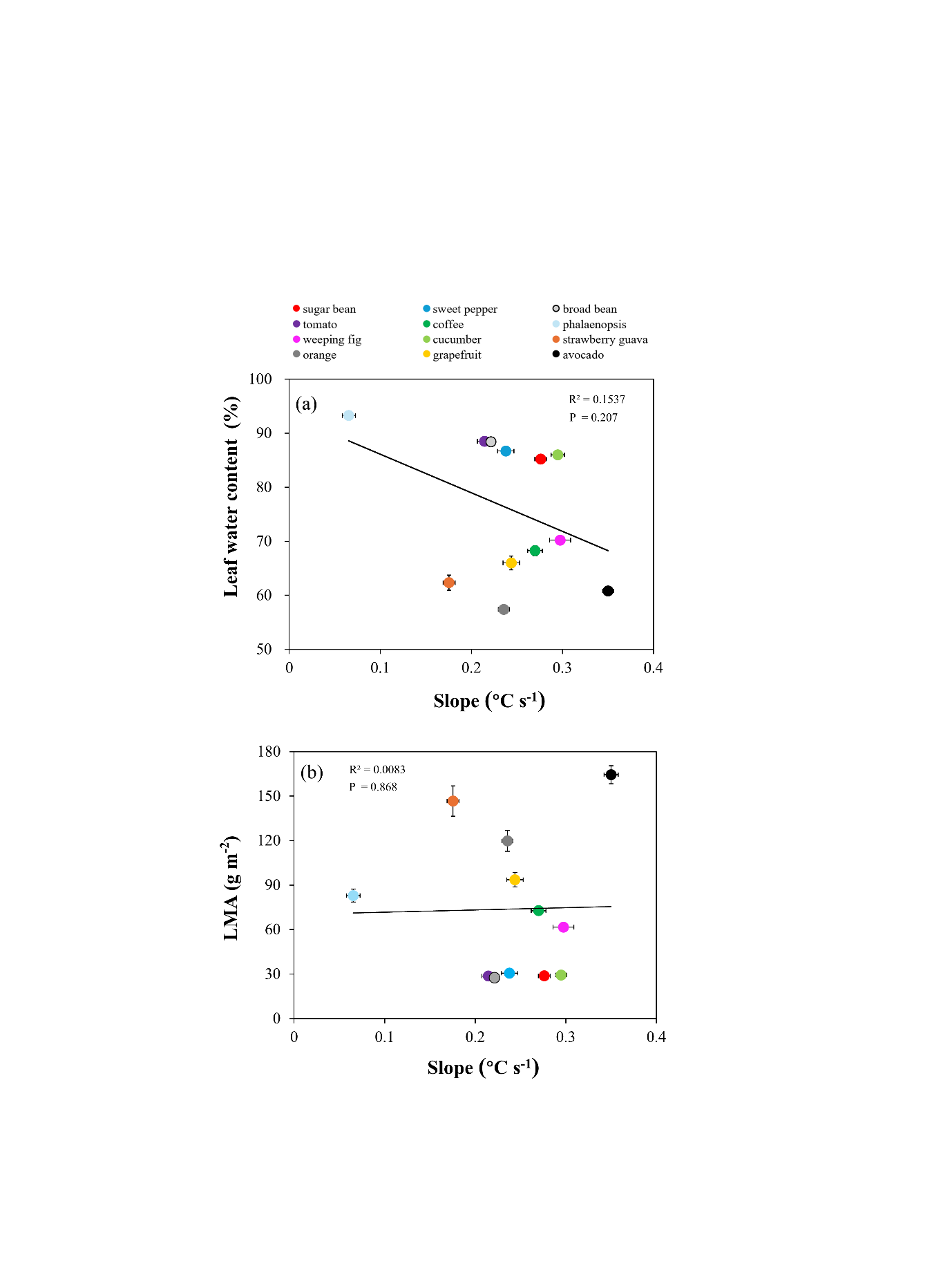


Figure S7. Relationships between the slope of leaf temperature increase (^°^C s^-1^) during high irradiance exposure and (a) leaf water content (%), and (b) LMA (g m^-2^) (means ± SE, n= 8). Due to data loss, pumpkin is not shown in Figure S7.
